# An integrated approach to health, wellbeing, and productivity at work: a design of a stepped wedge worksite intervention study

**DOI:** 10.1186/s12889-023-16014-x

**Published:** 2023-06-02

**Authors:** Mette Korshøj, Vivian Rueskov Poulsen, Margrethe Bordado Sköld, Sanna Koch Autrup, Brian Oldenburg, Ole Steen Mortensen

**Affiliations:** 1 Department of Occupational and Social Medicine, Hospital Holbæk, Gl. Ringstedvej 4B, 4300 Holbæk, Denmark; 2 Baker Heart and Diabetes Institute, Melbourne, VIC Australia; 3 School of Psychology and Public Health, La Trobe University, Melbourne, VIC Australia; 4 Section of Social Medicine, Department of Public Health, University of Copenhagen, Copenhagen, Denmark

**Keywords:** Occupational health and safety, Health promotion, Workplace health, Worker safety, Denmark, Musculoskeletal disorders, Mental health and wellbeing, Participatory approach, Organizational integration

## Abstract

**Background:**

Despite an intensive focus on workers’ health during recent decades, the prevalence of work-related diseases remains unchanged in Denmark and internationally. Therefore, USA and Australian researchers have initiated new paradigms for integration of health promotion, prevention of work-related disease, and organization of work. Inspired by the Australian WorkHealth Improvement Network program (WIN), this paper describes the background, design, intervention methodologies, and evaluation methods of an Integrated Approach to Health, Wellbeing, and Productivity at Work (ITASPA) intervention aiming to prevent work-related injuries and diseases and promote the health, safety, and wellbeing of the worker.

**Methods:**

Using a stepped wedge design, worksites will be enrolled at baseline and offered the intervention starting at different times. Data will be collected at baseline, before the off-set of the intervention, and after each implementation period. The effect evaluation will be based on a mixed-methods approach. The qualitative data are based on semi-structured interviews and focus groups. The quantitative data consists of questionnaires, anthropometrics, and resting blood pressure and will be analyzed based on the intention-to-treat principle in linear mixed models with random slope and intercept.

**Discussion:**

Integrated interventions are shown to increase overall health and safety at worksites more effectively and rapidly than more narrowly focused programs. Still, previous integrated interventions are lacking successful implementation. In ITASPA, the effects of the intervention is tested in a strong scientific mixed-methods design. Thus, the ITASPA project contributes to the knowledge about what characterizes a best practice for the implementation of integrated worksite interventions.

**Trial registration:**

ITASPA is retrospectively registered in Clinicaltrials.gov on May 19, 2023 (NCT05866978).

## Background

Despite the past decades’ increased focus on workers’ health, the Danish, as well as international prevalence of work-related diseases and injuries, remain stable [1, 2]. In Denmark, the most prevalent work-related diseases are musculoskeletal (MSDs) and mental disorders, e.g. work-related stress, depression, and anxiety [3], and the prevalence of work-related stress is increasing [4]. Such work-related diseases have negative consequences for the individual’s well-being and functional abilities and is a public health issue of major concern. Previously, worksite prevention of these conditions has mostly used single-faceted approaches addressing either health promotion (such as improving the general health and reducing chronic disease of the individual) or prevention of work-related risk factors for injuries or disorders [5]. However, separating activities addressing these two conditions are evidenced to limit their overall effectiveness [6]. Moreover, previous worksite interventions have lacked organizational integration [6]. To address the constant burden of work-related diseases and injuries and thereby ensure the safety and health of workers, leading health organizations have recommended better integration of the traditionally separate work health efforts [7–11]. However, little is known about best practice prevention [12], and more effective approaches to address and implement initiatives aiming to reduce work-related MSDs and mental disorders are needed.

The American Total Worker Health (TWH) [13] and the Australian WorkHealth Improvement Network (WIN) program [14, 15] are two examples of conceptualized integrated approaches towards health promotion and prevention of disease in work health interventions. TWH was introduced by NIOSH in 2011 with the overall aim of combining health promotion and prevention of work-related injuries and health risks factors in all work health interventions. Furthermore, TWH was based on the assumption that all contextual and work-related conditions affect the employee’s health behavior. The WIN concept operationalized the TWH approach and introduced an extended focus on systematic data collection and evaluation. Moreover, the WIN concept involved a collaborative element of knowledge-exchange across participating worksites and included ongoing adjustments of initiatives to improve the work health efforts.

Compared to the American work health system, the Australian system is more similar to the Danish, and the systematic approach and data collection in the WIN concept are possible to operationalize and have documented beneficial effects [15]. Therefore, our intervention ‘Integrated Approach to Health, Wellbeing, and Productivity at Work’ (ITASPA) is based on the concepts of WIN.

The aim of the ITASPA project is to investigate the effect of the WIN program in a Danish context. The hypothesis is that an integrated approach will improve the health of employees in terms of fewer MSDs, improved psychosocial wellbeing and workability, and strengthened occupational safety culture. Furthermore, the ITASPA project aims to examine the degree of implementation of the initiated initiatives and identify barriers and facilitating factors for implementation. This contributes to the knowledge about what characterizes best practices for the implementation of integrated worksite interventions.

## Methods

### Study design

**Fig. 1:**
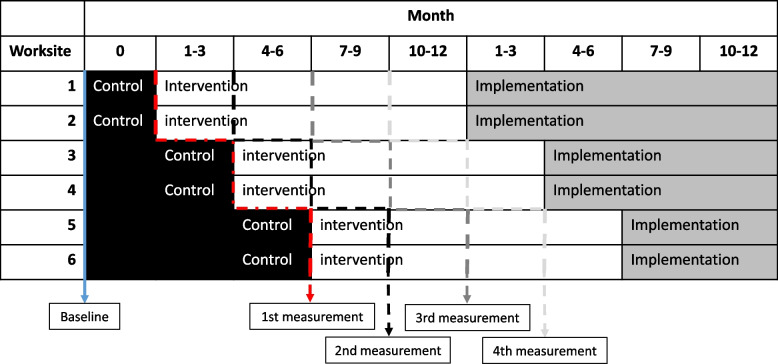
The stepped wedge design. All worksites are included at baseline. The black boxes illustrate periods where worksites will function as controls. The white boxes illustrate where worksites will function as intervention worksites and the grey boxes illustrate where the intervention is implemented and maintained

ITASPA is a worksite intervention, conducted in a stepped wedge design (Fig. 1) [16]. The stepped wedge design are assumed to increase the willingness to participate as it allows all workers to receive the intervention. The workers function as their own control and thereby, the stepped wedge design allows for an effect-evaluation, mimicking an RCT design.

The study was approved by the Danish data protection agency (journal number REG-034–2021) and the Ethics Committee for the regional capital in Denmark (journal number SJ-927) and will be conducted in accordance with the Helsinki declaration. The first version of the Clinicaltrials.gov registration for ITASPA is registered on May 19, 2023 (NCT05866978).

### Recruitment

To increase the transferring of the WIN program to a broad variety of Danish worksites, both public and private worksites are aimed to be enrolled. The worksites will be enrolled on basis of their willingness to perform the ITASPA activities during paid worktime and participation in the scientific evaluation of the ITASPA project. Workers will be included in the scientific evaluation of the ITASPA project by the following criteria: aged 18 – 67 years old at baseline; employed at one of the enrolled worksites ≥ 20 h/week; not being pregnant; ability to understand and speak Danish or English; providing an informed signed consent prior to participation.

An information meeting at the worksites will be held before initiating the intervention. At the meeting, the background and aim of the ITASPA project are introduced. All workers will be invited to fill out a screening questionnaire in which they can choose to sign up for participation in the scientific evaluation.

### Power calculation

The sample size estimations were based on effect expectations in MSDs, on a scale ranging from 0–10, an expected variance of 2.1, an Alpha value of 0.05, and a power of 80%. The estimation showed a cluster size of 65 participants to identify a statistically significant effect, at a 5% level, of the worksite intervention.

### Development, planning, and preparation of the itaspa intervention

#### Organization of the project

**Fig. 2:**
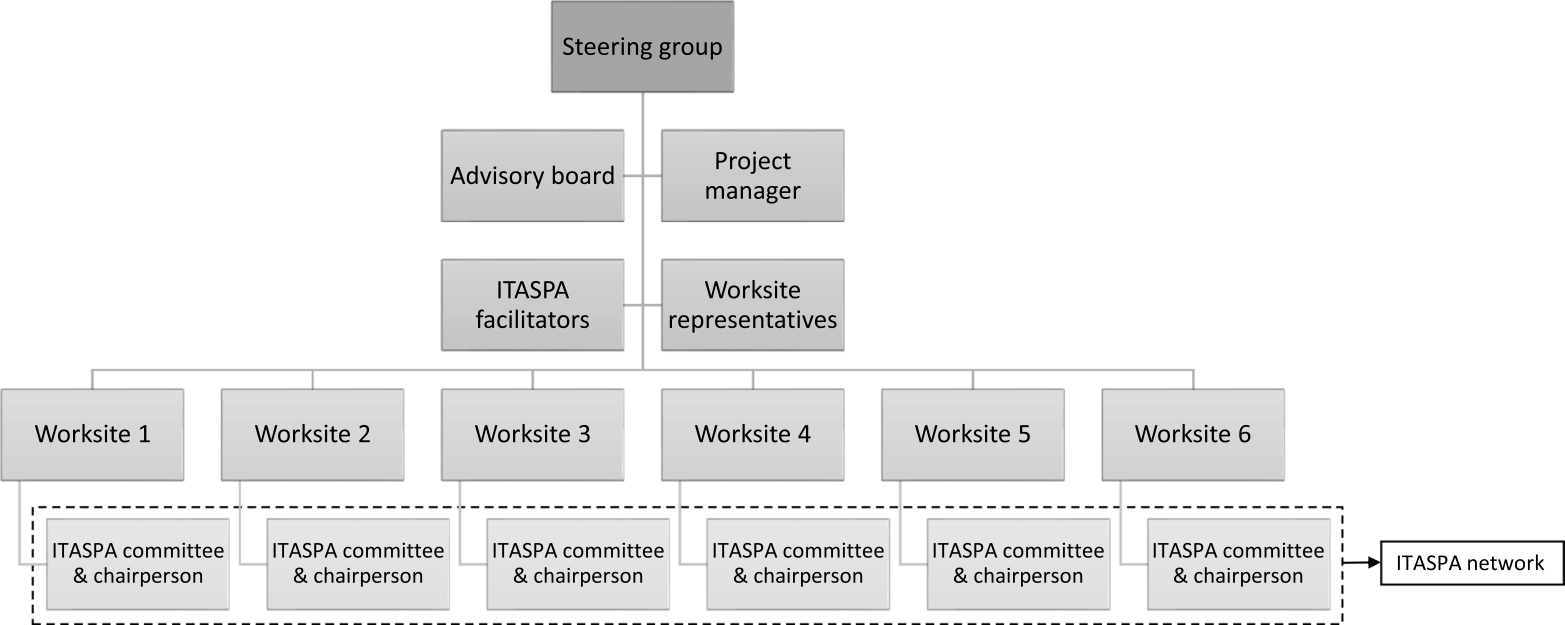
Flowchart of the organization of ITASPA

The ITASPA project will be organized by a steering group consisting of the ITASPA project managers, representatives from the senior management at the enrolled worksites, and the ITASPA facilitators from the Department of Occupational and Social Medicine at Holbæk Hospital, Denmark (Fig. 2). Moreover, the steering group includes an advisory board consisting of the developers of the WIN program and TWH concept. The advisory board will provide information about international experiences with integrated worksite interventions to assist the development of worksite health interventions in Denmark.

#### Development of ITASPA package of change

The steering group will collect and choose relevant tools and methodologies to establish a Package of Change. This package encompasses descriptions of the processes in ITASPA, evidenced examples of previous successful worksite interventions similar to ITASPA, and the present work environment policy, program, and practice at the enrolled worksites.

#### ITASPA committee at the worksite

At each worksite, an ITASPA committee will be established (Fig. 2). In accordance with an integrated approach, the members will represent the existing cooperative work environment fora, the workers, union and safety representatives, and relevant staff functions (HR, etc.). The worksites choose a chairperson for the ITASPA committee, who will be leading the interventions and act as the contact to the ITASPA facilitators. Ideally, the chairperson of the committee is a line manager who has knowledge of change management and communicates with both employees and senior management.

#### Education of the ITASPA committee

Before the offset of the intervention, the ITASPA committee will be educated by the ITASPA facilitators in the ITASPA concept and the Package of Change.

**Fig. 3:**
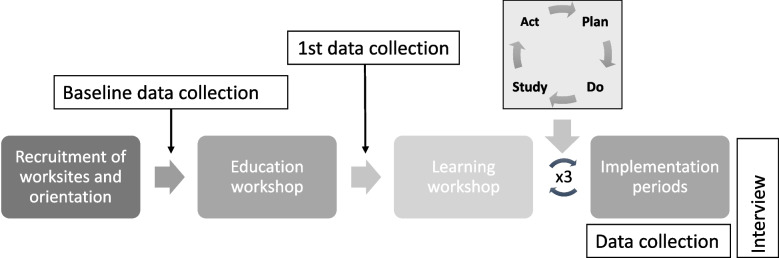
Process of the ITASPA intervention

The education session will be carried out as a workshop (education workshop, Fig. 3) at the enrolled worksites during paid work time. The workshop lasts 3 h and involves training in collaborative methodology, the models of improvements (e.g. the model of Change), the plan-do-study-act (PDSA) cycle, and the integrated concept of worksite intervention used in ITASPA [17]. A central theme for the education workshop is the Model of Improvement which consists of two parts. The first brainstorming part includes three key questions aiming to guide the assessment of which initiatives each worksite should focus on: 1) What are we trying to accomplish?, 2) How will we know that a change is an improvement?, and 3) What changes can we make that will result in an improvement?) [17]. The second part is the “test part”, which consist of the PDSA cycles:

• Plan: Thorough planning of the initiative, clarification of expectations of outcomes, and agreements on data collection.
• Do: Implementation of the planned actions as well as data collection.
• Study: Reflection on effect and learning, data analysis, and identification of unintended negative and positive consequences.
• Act: Assessment of either continuation of implementation, adjustments, or testing a new initiative.

The experiences from each cycle will systematically be transferred to the next. Thus, continuous improvements and/or adjustments will be conducted.

#### Workshops for choosing, planning, studying, and evaluating initiatives

In workshops (learning workshops, Fig. 3), the ITASPA committees will develop their initiatives based on their own experiences of their work environment. Methods used in the Australian WIN study [17] will guide the ITASPA committee in this process. Finally, success criteria will be set, and the committee will decide what data should be used to evaluate the effect of the initiative. All decisions will be documented by the ITASPA facilitators.

#### ITASPA committee network

Following the first implementation period, the ITASPA committees from all participating worksites will be invited to join a network. In this network, the ITASPA committees learn from each other, share ideas and give each other feedback. Each ITASPA committee will describe their worksite initiatives, the preliminary intended and unintended effects, and the facilitating factors and barriers for testing and implementation of their initiatives. The network meetings will be facilitated by the project manager and an ITASPA facilitator, who will lead the discussion and give feedback.

#### Periods of implementation (approximately 3 months)

Between the workshops the worksites will test and implement their initiatives, e.g. using the Model of Improvement from the ITASPA Package of Change. At each worksite, the ITASPA Facilitators participate in two of the ITASPA Committee’s meetings every implementation period to support the worksites and ensure an integrated approach.

### Data collection and study materials

#### Screening questionnaire

The screening questionnaire contains questions about job title, sex, age, country of birth, years lived in Denmark, job seniority, education, smoking, alcohol consumption, level of MSDs, safety culture at the worksite, psychosocial wellbeing, and diagnosis with one or more of the following diseases: asthma, allergy, diabetes, cardiovascular or skin diseases, and mental disorders.

#### Data collection at baseline and after the periods of implementation

**Fig. 4:**
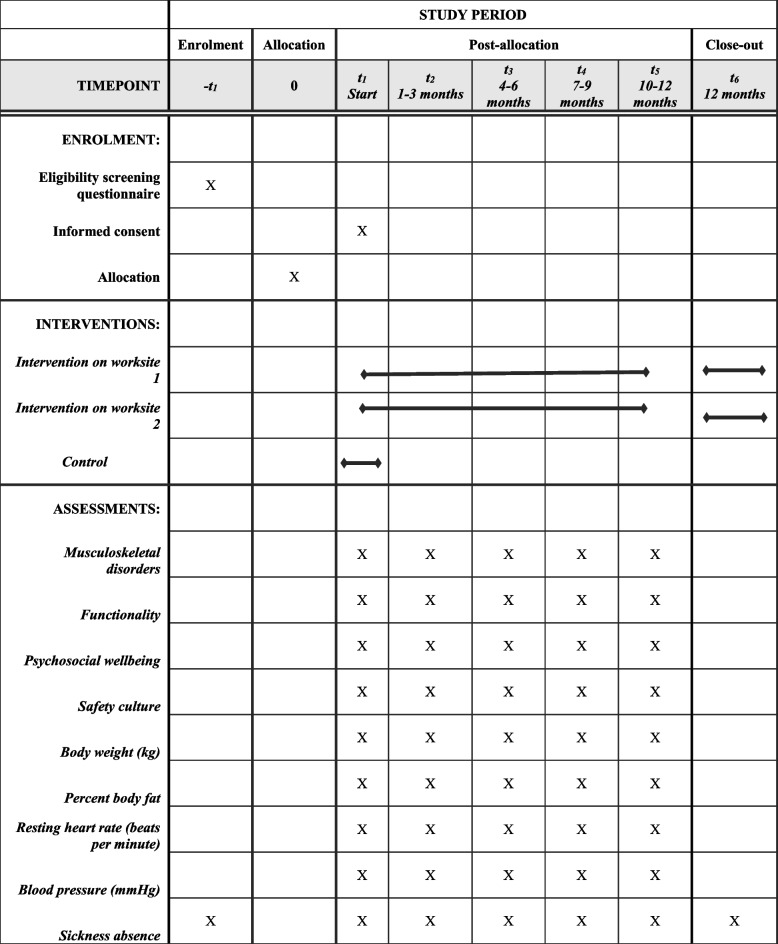
SPIRIT Flow diagram of the ITASPA intervention

The data collection will be conducted at baseline, before the first learning workshop, and after each period of implementation, approximately every third month throughout the year of scientific evaluation, summing up to five data collections in total (Fig. 4). The data collection will be conducted by the ITASPA facilitators at a health check encompassing a questionnaire-based interview and objective measurements.

The questionnaire-based interview includes questions about the level of physical activity in occupational and leisure time, eczema, and self-reported health. Functionality will be measured by the ICF-based Work Rehabilitation Questionnaire (WORQ) (https://myworq.org/). The Short Form of the Örebro Musculoskeletal Pain Screening Questionnaire will be used to measure MSDs [18]. Psychosocial wellbeing will be measured via the Health Survey SF-12 [19]. Finally, safety culture will be measured by the Nordic Occupational Safety Climate Questionnaire (NOSACQ-50) [20].

The objective measurements include body weight (kg), height (m), percent body fat, resting heart rate (beats per minute), and blood pressure (mmHg). Bodyweight, percent body fat, and height will be measured while the participant is wearing light clothes and no shoes. The estimated weight of clothes (1.5 kg) will be subtracted from body weight. Percent body fat will be estimated by bioelectric-impedance-analysis. Both body weight and percent body fat will be measured by a Segmental Body Composition Monitor, Innerscan V, BC545N (TANITA, produced in Japan). If participants have a pacemaker or are pregnant, the analysis of percent body fat will not be made. Body height will be measured on a mobile stadiometer Seca 213 (Seca, produced in China). Body mass index (BMI) will be estimated by the equation of BMI = (body weight (kg)/body height (m)^2^). Resting heart rate and blood pressure will be measured three times on the left arm after 15 min of sitting at rest using an Omron Model M3, automatic upper arm BP monitor (Omron healthcare, produced in Vietnam).

At baseline, and after the periods of implementation, we will collect data on sickness absence, staff turnover, and productivity combined with interviews with the chairperson of the ITASPA committee. These data will be collected for a financial evaluation of the overall ITASPA intervention at each worksite.

### Process and implementation evaluation

The implementation of the initiatives will be tracked by the research group using quantitative and qualitative methods, allowing for triangulation of data across methodologies [21].

The quantitative process tracking will be based on the data collected by the ITASPA committees during the implementation periods. Data will be used to monitor the fidelity and dose (the amount of intervention delivered) of the planned initiatives, following the principles of process evaluation [21].

The qualitative process tracking will be based on focus group interviews consisting of members of the ITASPA committee at each worksite after the second learning workshop and an individual semi-structured interview with the chairperson of the ITASPA committee midway through each implementation period. Data on implementation experiences will be collected and feedback on the effect of the chosen outcome, for the particular period of implementation, will be given. This has two purposes: 1) to collect data on factors facilitating and hindering the implementation of the initiatives, and 2) to give feedback to the worksites regarding the effect of the chosen outcome, based on the initiative in the ongoing period of implementation. The focus groups are directed by a semi-structured interview guide including questions about the specific interventions and reflections on intervention delivery, such as observations on contextual factors, barriers, and facilitating factors of intervention implementation [21]. Thus, the interviews will be tailored to the context and initiative at each worksite and provide input for the next period of implementation. Conversation objectives, topics, and materials discussed will be documented.

### Effect evaluation

Data from the health checks will be used to evaluate the effect of the initiatives after each implementation period. Preliminary descriptive analysis will be conducted and discussed with the ITASPA committee after each implementation period to ensure that only beneficial and effective initiatives are continued in the next implementation period and to guide the development of future initiatives.

### Statistical analysis

Descriptive analyses of baseline data and health check data will be conducted after the implementation periods. Frequencies and distribution of participants across variables will be reported (amount and percentages). Differences between baseline data and the follow-ups within and between participating worksites will be reported by the use of chi^2^ test for categorical variables and paired and independent sampled t-test for continuous variables. Furthermore, differences in baseline data between those who want to participate and those who do not want to participate will be analyzed. Where appropriate, results are presented as the mean and standard deviation (Mean ± SD) or visualized in graphs and histograms.

### Effect evaluation and analyses

The effect evaluation will be based on a mixed-methods approach, where questionnaire data on MSDs, psychosocial wellbeing, safety culture, and workability will be measured based on the questionnaire-based interviews. The quantitative data will be analyzed based on the intention-to-treat principle in linear mixed models with random slope and intercept. The repetitive measurements within and between participants will be accounted for by nesting participants in clusters at the worksite level. The intervention will be included as a categorical variable with two levels, comparing the intervention period with the control period as a fixed effect in the model. Intercorrelation of repeated measurements will be included in the models as a random effect. Participants missing data on exposure or outcome variables will be excluded from analyses. All analyses will be conducted in SAS version 9.4 and/or SPSS version 27.0.

### Evaluation of the implementation

Experiences from each interview midway through the periods of implementation will be analyzed in an overall analysis based on the Model of Knowledge to Action Cycle [22]. This analysis will be supplied by focus group interviews with the ITASPA committee chairpersons at the end of the intervention period.

### Ethical considerations

The implementation of the project within worksites depends on the consent of the top management. At learning workshops, worksites will be encouraged to share experiences with their work environment initiatives. During this process, company-sensitive information may be shared. Thus, in workshops, it will be pointed out that a duty of confidentiality is expected among companies.

Personal information will be obtained from the participants about safety at work, workability, MSD, and psychosocial well-being. Data will be stored and processed as prescribed by the Danish Data Protection Agency and will only be available to researchers involved in this study. Data will be anonymized in analyses and feedback to the worksite will be provided anonymously, and in groups of at least 10 participants. The ITASPA facilitators will discuss any unforeseen ethical aspects with the worksites if they experience any. In this context, the steering group and reference group will be involved as potential sparring partners.

## Discussion

The aim of the ITASPA project is to investigate the effects and implementation of the Australian WIN program adapted to the Danish context. The background, design, intervention methodologies, and evaluation methods have been described. Effective interventions aiming to reduce work-related MSDs and mental disorders remain to be established, and to this date only a few integrated worksite intervention studies have been conducted in a Danish context [23–25].

### Impacts of results

We hypothesize that an integrated approach will improve the health and wellbeing of workers and strengthen the occupational safety culture. Other international experiences show that comprehensive, integrated worker health programs linking health protection and health promotion activities increase overall health and safety at worksites more effectively and rapidly than more narrowly focused programs [6, 14]. For example, changes aimed at sleep deprivation as a safety issue have also shown improvements on health issues such as diabetes, obesity, and cardiovascular disease among workers [26]. Therefore, integrated approaches can create health improving changes that lies beyond what is traditionally categorized as occupational health and illness [6]. Thus, implementing an integrated worksite intervention in a Danish context has potential to address important public health issues by using the worksite as arena.

In ITASPA, activities are integrated and adapted into existing core tasks, health promotion/disease prevention strategies, and organizational structures at the worksite. Hence, initiatives developed in ITASPA can be integrated across organizational structures. Furthermore, ITASPA involves employees and managers in the development and implementation of initiatives. Such a participatory approach in interventions for workplace improvements has previously shown to effectively improve health conditions such as mental health [27]. However, earlier worksite interventions have lacked implementation and comprehensive evaluation. As ITASPA will evaluate the effect and process of an integrated worksite intervention in a strong scientific mixed-methods design, ITASPA will provide knowledge about the barriers and facilitating factors for the implementation. Thus, ITASPA has the potential to contribute to the knowledge about what characterizes a best practice for the implementation of integrated worksite interventions in a Danish context.

### Strengths and limitations

Even though RCTs are the golden standard for intervention studies, it is not always practically possible, nor feasible, to have a control group of employees working at worksites that will not be affected by the intervention. However, the stepped wedged design will function as an RCT as it allows for comparison of control and intervention effects within the same environment, without increasing the risk of contamination between the intervention and control group, which reduces the risk of bias. Moreover, a stepped wedge design can prevent ethical objections arising from withholding an intervention anticipated to be beneficial [28]. In addition, a stepped wedge design will overcome issues with impaired organizational commitment and disappointed participants in the control group since all participants will be offered the intervention, and thus increases the participants’ obligations to the project [25]. Yet, the stepped wedge design still allows a sound scientific evaluation in a RCT design and address important ethical considerations, thereby increasing the possibility of a high number of participants and compliance, leading to stronger estimates in our data analysis.

Some limitations of the intervention design also deserve to be mentioned. As all employees will receive the intervention, it is not possible to blind participants or those involved in delivering the intervention. Hence, information bias may appear, particularly when outcomes are subjective. E.g. it is possible that participants are likely to report more positive health outcomes due to extended awareness of changes in these conditions after having received the intervention, leading to stronger effect-estimates of the intervention [28].

Furthermore, selection bias may challenge the generalizability of the results: it is plausible that healthy workers are more likely to stay at work longer than those who are injured or made sick from work [29] (healthy worker selection bias). This may reduce the potential to identify adverse health effects from work, leading to weaker estimates of the effect of risk factors on the health outcomes. However, by using a screening questionnaire we can examine if those who want to participate are different from those who do not want to participate.

The companies are randomly sampled, allowing for enrollment of diverse industries. Thus, we are able to investigate if the integrated approach can be translated and be successful in different lines of industries. Previous findings from worksite intervention studies show that the beneficial effects of an integrated worksite intervention are seen across different industries such as cleaning services [30], healthcare [16], construction, and manufacturing [23]. Moreover, worksite intervention studies have shown significant beneficial changes among employees in different countries [14]. These results suggest that integrated approaches in worksite intervention programs can be relevant and implemented in diverse industries and countries.

## Data Availability

The datasets that will be used and/or analyzed during the current study are available from the corresponding author on reasonable request.

## Abbreviations

ITASPA: Integrated Approach to Health, Wellbeing, and Productivity at Work
WIN: WorkHealth Improvement Network
TWH: Total Worker Health
MSDs: Musculoskeletal Disorders
WORQ: Work Rehabilitation Questionnaire
NOSACQ-50: Nordic Occupational Safety Climate Questionnaire
BMI: Body Mass Index
PDSA: Plan-Do-Study-Act
